# Improved leptin sensitivity and increased soluble leptin receptor concentrations may underlie the additive effects of combining PYY [[1], [2], [3], [4], [5], [6], [7], [8], [9], [10], [11], [12], [13], [14], [15], [16], [17], [18], [19], [20], [21], [22], [23], [24], [25], [26], [27], [28], [29], [30], [31], [32], [33], [34]] and exendin-4 on body weight lowering in diet-induced obese mice

**DOI:** 10.1016/j.heliyon.2024.e32009

**Published:** 2024-06-03

**Authors:** Birgitte S. Wulff, Rune Ehrenreich Kuhre, Madhan Selvaraj, Jens F. Rehfeld, Kristoffer Niss, Johannes J. Fels, Secher Anna, Kirsten Raun, Marina Kjaergaard Gerstenberg

**Affiliations:** a Global Drug Discovery, Novo Nordisk A/S, 2760, Måløv, Denmark; b Translational Research, Global Translation, Novo Nordisk A/S, 2760 Måløv, Denmark; c Department of Clinical Biochemistry, Rigshospitalet, University of Copenhagen, DK-2100 Copenhagen, Denmark; d Biomarker Discovery, R&ED Digital Science and Innovation, Novo Nordisk A/S, 2760 Måløv, Denmark; e Research Bioanalysis, Global Research Technologies, Novo Nordisk A/S, 2760 Måløv, Denmark; f Translational Medicine, Global Translation, Novo Nordisk A/S, 2760 Måløv, Denmark

**Keywords:** Appetite, Area postrema, Body weight, Exendin-4, PYY(3–36), Leptin sensitivity, Soluble leptin receptor, Synergy

## Abstract

**Objective:**

Co-treatment with long acting PYY and the GLP-1 receptor agonists has potential as an efficient obesity treatment. This study investigates whether the mechanisms behind additive reduction of food intake and weight loss depends on complementary effects in brain areas regulating food intake and if restoration of leptin sensitivity is involved.

**Methods:**

Diet-induced obese (DIO) mice were co-treated with PYY(3–36) and exendin-4 (Ex4, GLP-1R agonist) for 14 days using minipumps. Leptin responsiveness was evaluated by measuring food intake and body weight after leptin injection, and gene expression profile was investigated in various of brain regions and liver.

**Results:**

We show that weight loss associated with co-treatment of PYY(3–36) and Ex4 and Ex4 mono-treatment in DIO mice increased expression of several genes in area postrema (AP) known to be involved in appetite regulation and *Cart, Pdyn, Bdnf* and *Klb* were synergistically upregulated by the co-treatment. The upregulations were independent of weight loss, as shown by inclusion of a weight matched control. Moreover, PYY(3–36) and Ex4 co-treatment resulted in synergistically upregulated plasma concentrations of soluble leptin receptor (SLR) and improved sensitivity to exogenous leptin demonstrated by food intake lowering.

**Conclusion:**

The study results suggest that synergistic upregulation of appetite-regulating genes in AP and improved leptin sensitivity are important mediators for the additive weight loss resulting from PYY and Ex4 co-treatment.

## Highlights

• Co-dosing with PYY(3–36) and Ex4 to DIO mice resulted in:
• Synergistic upregulation of SLR in plasma and hepatic *Lepra* expression.
• Increased leptin responsiveness demonstrated by food intake lowering.
• Synergistic upregulation of genes in area postrema involved in energy balance.

## Abbreviation list

Agrp: Agouti-related peptide
AP: Area postrema
ARH: Arcuate hypothalamic nucleus
BAT: Brown adipose tissue
BDNF: Brain derived neurotrophic factor
CART: Cocaine- and amphetamine-regulated transcript
CEA: Central amygdala nucleus
CGRP: Calcitonin gene related peptide
CRH: Corticotrophin releasing hormone
CSF: Cerebrospinal fluid
DMH: Dorsomedial nucleus of the hypothalamus
DVC: Dorsal vagal complex
EX4: Exendin-4
FFA: Free fatty acid
FR: Food restriction
HFD: High fat diet
GLP-1: Glucagon like peptide 1
GLP-1R: Glucagon like peptide 1 receptor
KLB: Beta klotho
LCM: Laser capture microdissection
LEPRA: Leptin receptor, subunit a
LEPRB: Leptin receptor, subunit b
MC4R: Melanocortin 4 receptor
NPY: Neuropeptide Y
NRS: Nucleus of the solitary tract
NTSR1: Neurotensin receptor 1
PB: Parabrachial nucleus
PDYN: Prodynorphin
POMC: Pro-opiomelanocortin
PPARGC1A: Peroxisome proliferator-activated receptor gamma coactivator 1 alpha
PVH: Periventricular hypothalamic nucleus
PYY(3–36): Peptide YY(3-36)
QMR: Quantitative magnetic resonance
RIN: RNA integrity number
SEM: Standard errors of mean
SLR: Soluble leptin receptor
TG: Triglyceride
TH: Tyrosine hydroxylase
VEH: Vehicle
WM: Weight matched
Y2R: NPY subtype 2 receptor

## 1 Introduction

Peptide YY(3–36) (PYY(3–36)) and glucagon-like peptide-1 (GLP-1) are gut hormones co-secreted from intestinal L-cells in the distal gut in response to food intake to mediate post-prandial satiety and lower blood glucose concentration. Co-dosing with long acting PYY(3–36) and the GLP-1 receptor agonists has potential as an efficient obesity treatment as food intake and body weight is synergistically or at least additively reduced in animal models [[1], [2], [3], [4], [5], [35], [36]]. Also in humans co-dosing of native PYY(3–36) and GLP-1 have shown synergistic reduction in food intake [5]. However, the exact mechanisms behind the additive/synergistic effect on food intake and body weight loss are unknown. Energy homeostasis is tightly regulated by hypothalamic regions and by the hindbrain, which both integrate signals from the periphery and different parts of the brain to exert inhibitory or excitatory effects on neurons regulating food intake and energy expenditure [6,7]. In the brain, PYY(3–36) and GLP-1 target different neuron populations through receptor binding to their cognate receptors Y2R and GLP-1R, respectively. Following receptor binding, PYY(3–36) appears to have a neuronal inhibitory effect [8,9] whereas GLP-1 mainly has direct excitatory effect on neuron activity [10]. In normal weight mice, synergistically enhanced neuronal activation has been shown for the satiety pathway from the area postrema (AP)/nucleus of the solitary tract (NTS) over the parabrachial nucleus (PB) to the central nucleus of the amygdala (CeA) as well as in the mediobasal arcuate hypothalamus (ARH) after acute co-dosing with PYY(3–36) and Ex4 [4]. In this study, the results suggest that PYY(3–36) potentiates the effect of GLP-1 through lowering of inhibitory signals thereby enhancing the neuronal activation important to drive the additional weight loss when PYY(3–36) and GLP-1 are co-dosed.

In addition to direct targeting of relevant appetite-regulating nuclei in the brain, restoring sensitivity to the adipose secreted hormone leptin is also of interest in the treatment of obesity [[11], [12], [13]]. In regulation of energy balance, leptin acts to lower appetite and increase energy expenditure. Many individuals with obesity exhibit increased circulating concentrations of leptin without significant effect on energy balance. Instead, these individuals have developed hyperphagia, demonstrating a reduced responsiveness to leptin [14,15]. Also, a lowering in circulating concentrations of soluble leptin receptor (SLR) is implicated in human obesity [16,17] but has received limited attention. In humans, SLR is released into circulation through proteolytic cleavage of the extracellular domain of the leptin receptor and takes place constitutively but can also be induced by various stimuli such as fasting and caloric restriction [18,19]. Nevertheless, SLR binds free circulating leptin to prevent its action but it also acts to extent leptins half-life and increase the sensitivity to leptin [20]. Whether leptin sensitivity is improved after weight loss is highly debated [21,22], and the contribution of SLR in this regard remains to be elucidated. Several studies using weight loss-inducing peptides such as amylin, fibroblast growth factor 21 (FGF21) and Ex4 in rodent models, suggests that leptin sensitivity is improved and that SLR may play a role for this improvement [12,13,23,24]. Furthermore, increased leptin responsiveness in the brain may also affect appetite regulating genes and modulation of neuronal activity in the hypothalamic regions [11,[25], [26], [27], [28]]. Thus, in addition to the direct CNS targeted effect by appetite-regulating hormones, the effect of improved leptin sensitivity may likely act in a greater orchestra to control energy balance and lower body weight. In the present study, the aim was to investigate leptin responsiveness and SLR, as well as gene expression profile in different brain regions in DIO mice after weight loss induced by co-treatment with PYY(3–36) and Ex4 to understand potential complementary mechanisms. We investigated the following brain regions guided by cfos immunoreactivity after acute co-dosing of PYY(3–36) and Ex4 [4]; central amygdala (CeA), parabrachial nucleus (PB), ARH, AP, NTS, paraventricular nucleus (PVN), and dorsomedial hypothalamus (DMH).

## 2 Methodology

### 2.1 Experimental animals

The experimental procedures in the present study were approved by the Animal Experiments Inspectorate (2005/561–989) under Danish Ministry of Justice and were performed in accordance with the Danish Animal Experimentations Act. The study was performed at the Laboratory Animal Unit at Novo Nordisk A/S, Måløv, Denmark.

### 2.2 Study design

Twenty-one weeks old obese C57BL/6J male mice were obtained from Jackson laboratory (California, US). Four days after arrival the mice were single housed following 12/12 h light:dark rhythm with lights on at 6 a.m. The obese mice shifted diet from 60 % HFD to 45 % HFD (Research Diets # RD12451, New Brunswick NJ, USA) 8 days after arrival. All mice had free access to water. Eight days prior to study start, the mice were allocated into groups according to body weight and body fat mass to minimize statistical variations between the groups. At study start, the mice were 28 weeks old. Sixty obese mice with an average body weight of 40.7 g were included (n = 12/group). At day 0, Alzet mini-pumps with compound solution were implanted in the obese mice. The mice received daily Rimadyl (5 mg/kg) up to 3 days after the surgery. The dose volume per mini-pump was 12 μl/day. PYY(3–36) was dissolved in 20 mM acetate buffer at an of pH 3.0 and Ex4 was dissolved in 50 mM Natrium-acetat, 0.05 % tween 80, 2.0 % glycerol at pH 4.5. The vehicle dosed mice were dosed with vehicle buffers for both PYY(3–36) and Ex4, mice in the PYY(3–36) group were dosed with PYY(3–36) (300 nmol/kg) and vehicle buffer for Ex4, mice in Ex4 group were dosed with Ex4 (5 nmol/kg) and vehicle buffer for PYY(3–36) and mice in the combination group were dosed with PYY(3–36) (300 nmol/kg) and Ex4 (5 nmol/kg). The compounds were produced, purified and formulated at Novo Nordisk A/S (Måløv, Denmark). After treatment period, the remaining compound solution in the osmotic mini-pump was collected and analysed for peptide concentrations to assess stabilities. No differences in dose concentration were found prior to study start compared to end of the study (data not shown). At study termination, at day 15, food was removed at 6 a.m., start of light cycle, and take down was started 4 h later. Take down lasted for 2 h and mice were sacrificed in random order to ensure that fasting time was matched between groups. Mice being weight matched (to the combination treatment group) were food restricted (FR) by daily adjustment of food supply. Food restriction regime was: Day 1–3, 70 % FR, Day 4–5: 50 % FR, Day 6; 70 % FR, Day 7–9; 50 % FR, Day 10; 20 % FR (day of leptin challenge), Day 11–13; 40 % FR, Day 14; 30 % FR.

### 2.3 Alzet osmotic mini-pump preparation and implantation

The Alzet osmotic mini-pump model 2002 was used (infusion rate 0.5 μl/hr, reservoir volume 200 μl, 14 days capacity). The implantation of the osmotic mini-pump was performed under aseptic conditions. The mice were treated with Rimadyl (5 mg/kg) administered 30 min prior to being anaesthetized with isoflourane. The skin in the scapulae was cleaned with chlor-hexidine 0.5 % in 70 % ethanol and an incision was made and a pocket created by blunt dissection. The pump was placed in the pocket with the exit-port first. The incision was closed with two wound clips.

### 2.4 Food intake and body weight measurement

Food intake and body weight were measured daily by manual weighing. The weight match group were food restricted to weight match the group treated with PYY(3–36) and Ex4 in combination.

### 2.5 Body fat measurements

Body fat mass was determined by the Echo Medical System QMR scanner (EchoMRI 2004, Houston, TX, USA) as previously described [29].

### 2.6 Leptin challenge

Four days prior to the leptin injection, mice were mock handled to accustom them to s.c. injection at the specific time point. On the experimental day, the mice received a s.c. injection 2 h before onset of dark phase. Half of the mice within each group were injected with a recombinant met-human leptin analogue (3 mg/kg, Novo Nordisk A/S, Måløv, Denmark) whereas the other half received vehicle (same buffer as used for dissolving leptin). Leptin was dissolved in 8 mM phosphate with 19 mg/ml propylene glycol at pH 8.15. Injection volume was 2 ml/kg. Food intake and body weight was measured 4 h (expected max effect of leptin) and 20 h after dosing.

### 2.7 Plasma and tissue extraction at termination

The termination of the mice took place 4 h after onset of light ∼10 a.m. The mice were anaesthetized with isoflurane followed by retro-orbital puncture for blood sampling. Subsequently the mice were decapitated. The whole brain was frozen in a 2-methyl-buthane bath at −30 °C (approximately 30 s) and was stored at −80 °C. Liver biopsy from the left lateral lobe was stored in RNAlater for 5 days at 4 °C and then stored at −80 °C.

### 2.8 Plasma leptin and soluble leptin receptor (SLR) analysis

Plasma leptin and SLR were analysed at study termination. Leptin was quantified by a custom developed Mesoscale (MSD) multiplex assay, which is a multiarray assay with electrochemiluminescence readout. The MSD antibody measure exclusively freely available leptin with a reported lower limit of quantitation of 1.8 pM. Soluble leptin receptor was analysed by a mouse leptin receptor picokine ELISA kit (BOSTER biological technology, Cat # EK0440).

### 2.9 Cryo cut and Laser capture microdissection (LCM)

Frozen brains sections were stereologically sampled under RNA protective conditions on a cryostat. Coronal sections (20 μm) were placed on pre-cooled pen foil covered glass slides (Leica Microsystems). Four series of brain sections, defined by Paxinos & Watson” The Mouse Brain in sterotaxic coordinates, 4th Edition”, were collected; 1) PVH (incl. CeA) app. from bregma −0.34 mm to −1.23 mm; ARH (incl. CeA, DMH) app. bregma −1.23 mm to −2.53 mm; PB) app. bregma −5.07 mm to −5.41 mm; 4) AP (incl. NTS) app. bregma −7.31 mm to −7.67 mm. Following sectioning, the slides were stored at −80 °C until further processing. Prior to LCM, sections were thawed for ∼3 min at 4 °C. Next, the sections were stained for 4 min at 4 °C in a 0.1 % cresyl violet acetate solution (Sigma-Aldrich) dissolved in 70 % ethanol. Sections were next dehydrated in a graded ethanol series and air dried for ∼2 min in a fume hood. The relevant brain regions were identified and collected using a Leica LMD7 (Leica Microsystems). The brain regions were selected based on increased neuronal activity observed after acute co-dosing with PYY(3–36) and Ex4 in lean mice [4] and due to well-established neurocircuits involved in the regulation of food intake and energy expenditure.

### 2.10 RNA extraction and cDNA synthesis

For the brain regions, the RNeasy Plus Kit (Qiagen) was used for cell lysis and RNA extraction, following provided instructions. Following LCM, the remaining tissue from at least one series per animal was scraped off and used for assesment of RNA quality using an Agilent 2100 Bioanalyzer (Agilent Technologies) using the RNA 6000 Nano Kit. The tissue with an RNA Integrity Number (RIN) ≥6.7 was used for analysis. One cDNA replicate was made and stored at −80 °C until use.

### 2.11 Gene expression

The selected genes were guided by available RNA-sequencing data from in house studies (Novo Nordisk A/S) on the presumption of differential gene expression between the groups. The genes of interest in the brain were focussed on regulation of appetite, energy expenditure and neuronal communication. High-throughput qPCR was conducted with the Biomark HD system (Fluidigm) on a 96.96 IFC chip for all brain regions as described in Keinicke et al. [30]. SYBR-green based qPCR and conventional TaqMan primer sequence were used for the liver gene expression of *Lepra* (see Ref. [30]). Delta-delta CT values were used, and an average of the selected reference genes was used to calculate the relative gene expression profile. The selected reference genes included were (*Gapdh, Ppib, Tbp, Ywhaz, Ppia, Rps 18*), and all were stabile expressed in brain regions and liver.

### 2.12 Data analysis

Gene expression data from Biomark HD system obtained from the brain regions was analysed in Qlucore program using multigroup comparison with the p-value below 0.01 was included. The remaining pharmacodynamics data were analysed, and associated graphs were created in GraphPad Prism version 7.00 (GraphPad Software Inc, La Jolla, CA, USA). The statistical analysis was performed by a two-way ANOVA followed by a Turkeys’ multiple comparisons test to test for significance between groups and for synergism [31]. Data are presented as means ± SEM. A p-value of 0.05 or less was considered statistically significant.

## 3 Results

### 3.1 Body weight, fat mass and food intake

**Fig. 1:**
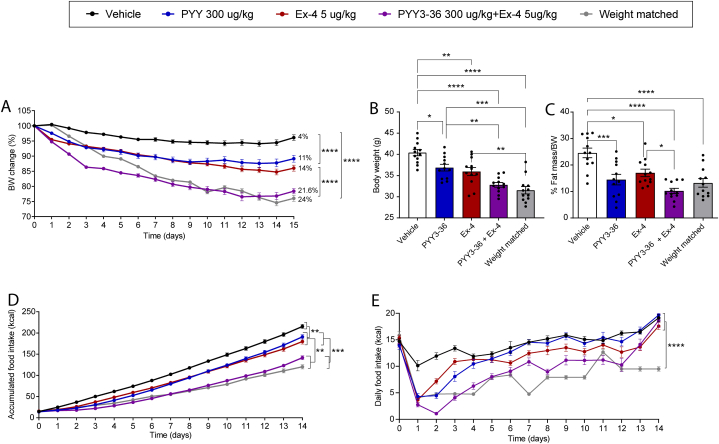
**PYY(3**–**36) and Ex4 in combination additively reduces body weight and accumulated food intake during 15 days of treatment.** A) body weight change (expressed in % relative to weight at day 0), B) body weights at day 15 (g), C): %fat mass/BW at 15 days after treatment, D) accumulated food intake (kcal), E) daily food intake (kcal). Data are expressed as means ± SEM, n = 12. Statistical significance was assessed by two-way ANOVA followed (A, D and E) or by one-way ANOVA (C and D). In both cases, ANOVA test's were followed by Tukey's multiple comparisons test. *p < 0.05, **p < 0.01, and ***p < 0.001.

Co-dosing of DIO mice with PYY(3–36) (300 nmol/kg) and Ex4 (5 nmol/kg) resulted in ∼21.5 % body weight reduction compared to vehicle, while mice being weight matched (WM) had body weight reduced with ∼24 %. Dosing with PYY(3–36) (300 nmol/kg) reduced body weight with ∼11 %, while Ex4 (5 nmol/kg) resulted in ∼14 % body weight reduction (Fig. 1A). If expressed as absolute body weights (g), significant treatment effects were also evident (Fig. 1B). At end of study, body fat mass was significantly decreased by all treatments compared to vehicle treated mice (p < 0.05–0.0001), and combination treatment decreased fat mass more than Ex-4 treatment (p < 0.05, Fig. 1C). At day 14, accumulated food intake was significantly decreased by all treatments compared to the vehicle treated mice (p < 0.05–0.0001) and was decreased more by combination treatment compared to mono-treatments (Fig. 1D). Most of the inhibitory effects on food intake occurred within the first 5 days of treatment in case of the mono-treatments whereas the combination treatment had more a sustained inhibitory effect (Fig. 1E).

### 3.2 Leptin challenge

**Fig. 2:**
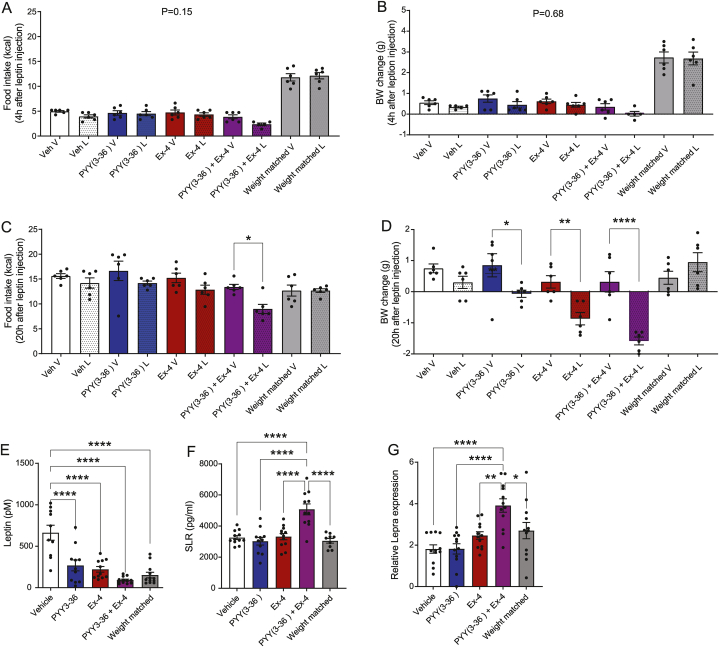
**PYY(3**–**36) and Ex4 in combination increases leptin sensitivity and increases the concentration of circulating soluble leptin receptor.** A-D) Accumulated food intake (kcal) and body weight change (g) 4h (A, B) and 20h (C, D) after leptin injection(5 mg/kg) at treatment day 10.E) Plasma leptin concentration (pM) at day 15, F: Plasma concentration of soluble leptin receptor (SLR) at day 15, and G: Hepatic *Lepra* expression at day 15. Data are expressed as means ± SEM, n = 12. Statistical significance was assessed by one-way ANOVA (E) or by two-way ANOVA followed (F and G). In both cases, ANOVA test's were followed Tukey's multiple comparisons test. In case of A-D, test of significance was restricted to comparing within group effects (vehicle and leptin treatment). *p < 0.05, **p < 0.01, and ***p < 0.001.

At treatment day 10, mice were dosed with leptin or leptin-vehicle immediately prior to the dark phase. Four hours after dosing, leptin had not resulted in lowering of food intake or body weight in any for the groups (P ≥ 0.15, Fig. 2A and B). However, 20 h after dosing, food intake was reduced with ∼30 % in the combination treatment group (P < 0.05), whereas food intake was unchanged in the remaining groups (Fig. 2C). Consistent with this, 20 h post dosing body weight was most pronouncedly reduced in the combination treatment group (P < 0.0001, Fig. 2D).

### 3.3 Plasma leptin and soluble leptin receptor (SLR)

Plasma leptin concentrations were reduced to more than half in all groups that lost weight compared to vehicle (Fig. 2E). Plasma SLR levels was significant increased by PYY(3–36) and Ex4 in combination compared to all groups (p < 0.0001) but was not increased between vehicle and remaining groups (Fig. 2F).

### 3.4 Liver

The expression level of *Lepra* was significantly higher in the PYY(3–36) and Ex4 combination group compared to all other groups (P < 0.05) whereas expression levels did not differ between remaining groups (Fig. 2G).

### 3.5 Gene expression profile in brain regions

**Fig. 3:**
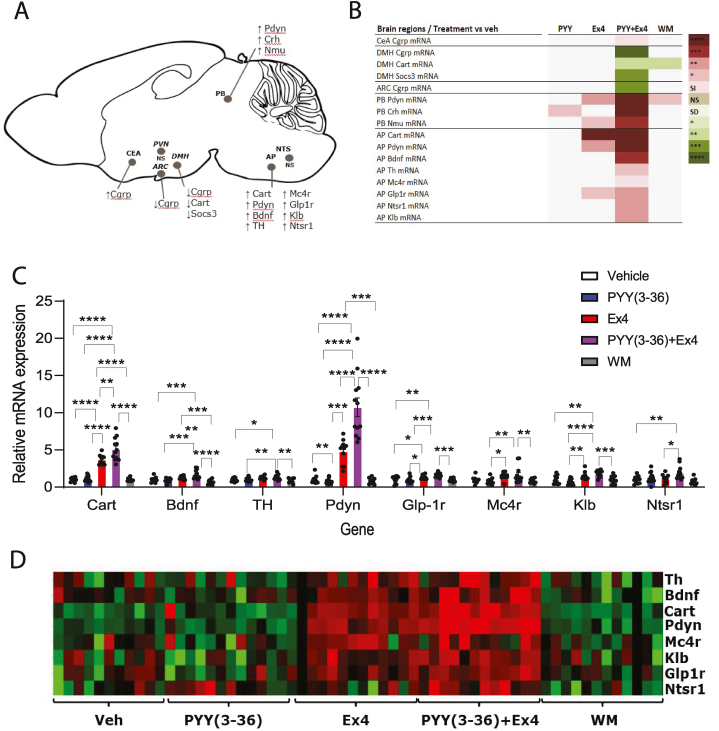
**Gene expression profile in the brain.** A) Overview of the analysed brain regions and regulated genes induced by combination of PYY(3–36) and Ex4, B) Significant regulated genes versus vehicle treatment in the different brain regions, C) Relative mRNA expression in AP to expression in vehicle group, D) Heatmap of regulated genes in AP. Data are expressed as means ± SEM, n = 12, analysed by two-way ANOVA followed by a Turkeys' multiple comparisons test to test for significance between groups.

The gene expression profiles from the different brain areas revealed that the AP exhibited the most significant regulation among the analysed brain regions (Fig. 3A). Mice treated with PYY(3–36) and Ex4 in combination and with Ex4 alone showed significant upregulation of *Cart, Pdyn* and *Glp-1r* in AP brain region compared to vehicle treated mice. Among these genes, *Cart, Pdyn, Bdnf* and *Klb* were synergistically increased by PYY(3–36) and Ex4 compared to Ex4. Furthermore, the combination of PYY(3–36) and Ex4 also resulted in a significant induction of *Th, Mc4R* and *Ntsr1* were also significant induced by the compared to vehicle (Fig. 3B–D). In the PB brain region, co-dosing with PYY(3–36) and Ex4 resulted in increased expression of *Crh* compared to all other groups as well as increased expression of *Pdyn* compared to mice dosed with vehicle and PYY(3–36). *Nmu* expression in PB was also increased in mice co-dosed with PYY(3–36) and Ex4 compared to vehicle, and WM. Mice treated with Ex4 alone had increased *Pdyn* and *Nmu* expression in PB compared to vehicle treated mice while WM had increased *Pdyn* expression compared to vehicle. Co-dosing with PYY(3–36) and Ex4 reduced *Cgrp* expression in CeA compared to WM, in ARH compared to vehicle and WM and in DMH compared to all groups. In DMH, PYY(3–36) and Ex4 in combination furthermore reduced *Cart* expression compared to vehicle and Ex4 dosed mice, and *Socs3* expression was reduced compared to vehicle, PYY(3–36), and Ex4 (Fig. 3B–D). None of the selected genes in NTS and PVH were affected by co-dosing with PYY(3–36) and Ex4 (Fig. 3A).

## 4 Discussion

In line with previous research in both rodents and humans [[1], [2], [3], [4], [5], [35], [36]], our current study demonstrates an additive reduction in body weight and accumulated food intake in DIO mice treated with PYY(3–36) and Ex4 for 15 days. Furthermore, leptin responsiveness is increased by the combination treatment only, as evidenced by a decrease in food intake 20 h after leptin dosing. Notably, at day 15, plasma concentrations of soluble leptin receptor (SLR) and hepatic *Lepra* expression was synergistically upregulated in the combination treatment group, indicating a potential contribution to the enhanced leptin sensitivity. Additionally, in the hindbrain's AP region, we observed increased expression of genes involved in appetite regulation, such as *Cart, Pdyn, Bdnf, Th, Glp-1r, Klb*, and *Ntsr1*. Among these genes, *Cart, Pdyn, Bdnf* and *Klb* were synergistically upregulated compared to mono treatment of Ex4. Importantly, these effects were not observed in the weight-matched control group, indicating that the responses were not a result of the weight loss per se. Our findings suggest that the combination of PYY(3–36) and Ex4 treatment exerts a beneficial impact on body weight, food intake, leptin responsiveness, and the expression of appetite-related genes, thereby providing promising insights for potential therapeutic interventions.

### 4.1 The combination of PYY(3–36) and Ex4 uniquely resulted in a synergistic upregulation of SLR, which may be associated with the improved leptin responsiveness observed in our study

In mice, soluble leptin receptor (SLR) can be generated through two mechanisms: the transcription of a specific isoform called LEPRE and the proteolytic shedding of membrane-anchored leptin receptor isoforms in the liver [18,19]. In contrast, in humans, SLR is exclusively generated via proteolytic shedding of membrane-anchored leptin receptor isoforms [[32], [33], [34]]. The difference in SLR generation between rodents and humans are reflected in our interpretation of our study results.

The synergistic increase in hepatic *Lepra* expression in mice co-dosed with PYY(3–36) and Ex4 suggest that this increase may be associated with enhanced shedding of hepatic *Lepra* and, consequently, the synergistic increase in SLR plasma concentrations. The concentration of SLR in plasma and its binding to leptin affects the availability of free leptin which plays a role in regulating appetite, metabolism, and energy balance. Lean humans have SLR that binds to the majority of circulating leptin [17,37], while individuals with obesity often have lower SLR concentrations, leading to increased plasma leptin levels and reduced responsiveness to leptin's effects [16,17]. In obese rodent models, genetically induced higher SLR concentrations have been observed to lower body weight by improving leptin responsiveness [20,38]. Our study corresponds with these findings, as increased SLR levels observed in mice co-dosed with PYY(3–36) and Ex4 were associated with an improved effect of exogenous leptin administration on reducing food intake and body weight. We suggest that the increase of SLR enhances leptin's availability and prolong the effect, as evidenced by the sustained response to leptin administration up to 20 h. Supported by the gene expression in the brain (discussed later), it implies an improved ability of the brain to sense the leptin signal. As a result, these findings suggest a more effective leptin-induced satiety response, potentially contributing to weight loss, and the preventing of overeating and subsequent weight regain. Nevertheless, one limitation of our study design was the lack of determination of SLR upregulation timing, preventing us from knowing the SLR levels at day 11. This requires further investigation to confirm. Furthermore, when treated separately with either PYY(3–36) or Ex4, there was some treatment response to leptin observed on body weight at 20 h while no effect at 4 h. Hence, the mono-therapy approach indicates an improvement in leptin sensitivity, while the combination therapy results in a synergistic effect on leptin sensitivity.

In the context of weight maintenance after weight loss, we and others (e.g., Rosenbaum et al. [22,39] speculate that reduced sensitivity to leptin might contribute to weight regain. Weight maintenance after caloric restriction and GLP-1-induced weight loss has been challenging [40]. Despite achieving normalized plasma leptin concentrations following caloric restricted weight loss, individuals often experience increased food seeking, reduced satiety response, and lower energy expenditure, suggesting a reduced responsiveness to leptin [22,39]. Some human trials involving caloric restriction and gastric sleeve procedures have investigated SLR levels and found them to be increased but not normalized [17,[41], [42], [43]] or unaffected [44]. In fasting condition SLR become upregulated presumably to prevent further reduction in food intake through leptin [18]. It's important to note that plasma leptin levels naturally drop after weight loss, corresponding to the reduction in fat mass and thus no need to decrease leptin levels as compared to fasting conditions where fat mass is unaffected. The lack of SLR normalization in caloric restricted weight loss could explain the increased food seeking and reduced energy expenditure observed in humans despite having normal leptin concentrations.

In our present study, we observed that mice losing weight through caloric restriction did either not show an increase in SLR levels, and leptin sensitivity was not improved. This finding aligns with the findings by Müller et al., where caloric-restricted mice, after a 28 % weight loss, did not further reduce body weight when treated with leptin. In contrast, mice with an equal amount of weight loss induced by Ex4 showed further body weight reduction when treated with leptin, suggesting an improved leptin responsiveness [12]. In our study, we also observed an effect of the leptin on body weight with Ex4 treatment alone at 20 h but not to the same extend as with combination treatment. The difference in leptin responsiveness with Ex4 between our study and that of Müller et al. could be attributed to the magnitude of weight loss (14 % vs. 28 %) and needs to be further addressed.

However, our next question is to understand why SLR becomes synergistically upregulated in combination therapy with PYY(3–36) and Ex4 and how this relates to the balance of leptin-SLR and improved leptin sensitivity. We also need to investigate whether this finding is specific to rodents or if it can be translated into humans. To date, there has been no clinical evidence addressing SLR levels induced by pharmacological weight loss treatments. Thus, we hope that this publication will contribute to fill this knowledge gap and include such measurements in clinical studies. The mechanisms driving the synergistic increase in hepatic *Lepra* expression and its potential shedding into SLR following pharmacological treatment have not been addressed yet. Interestingly, since neither the Y2 receptor nor the GLP1 receptor is expressed in the liver, this increase may occur via indirect mechanisms. Similar increase in hepatic *Lepr* expression with FGF21 monotherapy achieving 20 % weight loss and a synergistic upregulation in combination with GLP-1 have been observed in DIO mice [24,30]. One possible explanation for the increased *Lepra* expression could be mediated through pharmacological improved insulin signaling supported by leptin and insulin sensitivity interact [45] or centrally mediated. However, it's important to note that our current study did not specifically investigate insulin sensitivity but it is well known that PYY(3–36), GLP1 and FGF21 improve insulin sensitivity [[46], [47], [48], [49]].

Furthermore, studies in rodents have also suggested that SLR may improve metabolic health independently of leptin, as mice overexpressing human hepatic SLR showed lower body weight independently of leptin [38,50]. Our study's findings indicated that the loss of fat mass in all treatment groups correlated with reductions in leptin concentrations. The combination group, treated with PYY(3–36) and Ex4, did not show further reduction in leptin concentrations with SLR increase, suggesting that SLR may have effects independent of leptin. However, as both increased and reduced SLR concentrations can negatively impact leptin sensitivity, maintaining a well-balanced ratio of the leptin-SLR complex is crucial for the effectiveness of leptin and overall body weight homeostasis [51]. Nonetheless, further investigations are warranted, and essential to address both total SLR and free SLR levels, which were not measured in the present study.

Overall, the present study and existing literature highlight the importance of understanding the effect of SLR in the context of caloric restriction and pharmacologically induced weight loss and its potential translation into humans. Given the limited research focus on investigating SLR, we recommend measuring both total SLR and free SLR, as this would enhance the interpretation of leptin sensitivity and increase attention to its role in weight regulation.

### 4.2 Mice treated with Ex4 and the combination of PYY(3–36) and Ex4 exhibited significant gene regulation in the area postrema (AP), a brain region associated with energy balance

The gene expression profiles from the different brain areas revealed that the AP exhibited the most significant regulation among the analysed brain regions. This observation was expected, considering that the AP is a circumventricular organ with permeable microvasculature, allowing for access to humoral input. Additionally, its proximity to the dorsal vagal complex (DVC), which receives input from the vagus nerve, could contribute to its responsiveness. Bulk and single-cell RNA sequencing data also support the idea that genes in the AP region are affected by GLP-1 treatments [52]. Immunoreactivity analysis of cfos, a marker for neuronal activation, after co-dosing with PYY(3–36) and Ex4, further supports the notion that AP, along with the nucleus tractus solitarius (10.13039/501100003594NTS), exhibits the most intense neuronal activation [4]. This highlights the significant impact of the treatment on the AP region. Furthermore, both Y2R (Y2 receptor) and GLP-1R (GLP-1 receptor) are widely expressed throughout the brain [[53], [54], [55], [56]] and the vagus nerve [57]. Studies have shown that GLP-1 can bind to specific brain areas, such as the hypothalamic arcuate nucleus (10.13039/501100001036ARH), AP, and 10.13039/501100003594NTS, further supporting GLP-1 activity in these regions [54,58,59]. In the AP, mice treated with Ex4 alone and the combination of PYY(3–36) and Ex4 exhibited increased expression of *Cart, Pdyn,* and *Glp-1r*, with *Cart, Pdyn, Bdnf* and *Klb* showing synergistic upregulation. In addition, *Th, Mc4R* and *Ntsr1* were also significant induced by the combination of PYY(3–36) and Ex4 compared to vehicle. GLP-1R expression has been identified on CART neurons in ARH, glutaminergic neurons in the hindbrain, and TH neurons in both the hypothalamus and hindbrain [59], suggesting increased binding and activity to these neurons. PYY(3–36) binding has been shown to produce an inhibitory neuronal response, which may potentiate additional GLP-1R signaling by inhibiting neurons that negatively regulate GLP-1R neurons [4], thus explaining the synergistic upregulation of genes activated by Ex4. Another possible explanation for the increased gene expression could be the recruitment of different signaling pathways within the brain since PYY(3–36) and GLP-1 are known to act on different neurons [15,[60], [61], [62], [63]]. Furthermore, the increased gene expression may also be mediated by increased leptin responsiveness, as discussed below.

### 4.3 The enhancement of leptin sensitivity could potentially influence gene regulation in the AP

Previous studies have shown that LEPRB is co-expressed with CART, PDYN, and TH neurons [[64], [65], [66]]. As an example, in the ARH, CART expression is positively regulated by plasma leptin concentrations, resulting in increased CART expression and reduced food intake in DIO mice [67]. It is possible that a similar mechanism may also occur in the AP. Ablation of LEPRB in PDYN neurons in the hindbrain has been demonstrated to lead to decreased energy expenditure, highlighting the importance of the interaction between leptin and PDYN in body weight regulation [65]. Overall, LEPRB-expressing neurons in AP and NTS appear to play a crucial role in the regulation of energy balance [68].

Although daily food intake reached comparable levels to the vehicle group by the end of the study, the sustained effect of lowering body weight could be attributed to an increase in energy expenditure mediated by enhanced leptin sensitivity. The upregulation of neurons expressing LEPRB suggests that improved leptin effectiveness in the hindbrain could be a contributing factor. Moreover, the increased expression of BDNF protein in the DVC brain region, as induced by both single and repeated peripheral leptin treatments in mice [69], indicates that BDNF expression is influenced by leptin. This synergistic upregulation of *Bdnf* expression in our study may also contribute to the improved leptin responsiveness and underlie the additive effects of combining PYY(3–36) and Ex4. Notably, in the same publication, it was suggested that BDNF expression mediates the synergy between leptin and cholecystokinin (CCK) in reducing food intake and body weight through melanocortin signaling [[69], [70], [71]]. In the present study *Mc4R* (melanocortin 4 receptor) were slightly upregulated in response to the combination of PYY(3–36) and Ex4, which could indicate a need for further analysis of CCK and the melanocortin system in GLP-1 induced weight loss. Also, because studies in mice suggest that CCK might be crucial for GLP-1's satiety action [72,73]. Lastly, the synergistic upregulation of *Klb* expression observed in the present study could point towards a role for FGF21 signaling in AP contributing to the additive effect on weight loss and leptin sensitivity. This is based on the increased hepatic Lepr expression with FGF21 alone and in combination with GLP-1 in previous studies [30,74].

### 4.4 The combination of PYY(3–36) and Ex4 has limited impact on genes related to energy balance in other brain regions

In the PB brain region, the combination of PYY(3–36) and Ex4 led to increased expression of *Crh*, *Pdyn*, and *Nmu*. It is not clear whether this increase is influenced by enhanced gene expression and neuronal projections from AP. However, it is known that GLP-1R responsive TH neurons innervate PB [75], and the synergistic upregulation of TH in AP suggests a possible role in this brain region. The specific role of the increased expression of *Crh, Pdyn*, and *Nmu* in PB is not fully understood in our study, but they are likely to contribute to the body weight-lowering effect. For instance, the increased *Pdyn* expression could indicate enhanced thermogenesis, as Pdyn-positive neurons in PB have been associated with increased thermo-sensation [76]. The role of *Nmu* in PB is unknown, but administration of NMU and overexpression of NMU in mice have been shown to promote leanness through a reduction in food intake and increased energy expenditure [77]. CRH has been linked to stress response when expressed in the paraventricular nucleus (PVN), but in other brain regions, CRH appears to be important for regulating food intake and energy expenditure. Additionally, injection of a CRH antagonist has been demonstrated to prevent the anorectic effects of leptin, indicating potential interaction between leptin and CRH [78]. In the CeA brain region, the increased expression of *Cgrp* may originate from PB CGRP projections [79]. Activation of CGRP neurons in PB has been associated with malaise and food intake reduction [80,81], but CGRP is also implicated in nociception [79]. Therefore, the increased *Cgrp* expression in CeA may be linked to PB, as its CGRP neurons project to CeA. Within the DMH region of the brain, the combination of PYY(3–36) and Ex4 resulted in reduced *Cart* and *Socs3* expression. This reduced expression of *Cart* in DMH may indicate restored CART expression in mice co-dosed with PYY(3–36) and Ex4. CART is highly expressed in feeding-related areas [66] and has both anorexigenic and orexigenic properties depending on its location in the brain [82,83]. For instance, in hypothalamus of DIO mice, CART expression is reduced in ARH and PVH while upregulated in DMH and LHA [84]. The different expression profiles of CART in DMH and AP align well with the anorexigenic and orexigenic properties of CART related to weight loss in our study [67,85,86]. The reduced *Socs3* expression in DMH could be an indicator of increased leptin signaling [87,88], contributing to the weight-lowering effect [89,90]. Although SOCS3 is not exclusive to leptin signaling, it has been associated with weight regulation. In contrast, no significant regulations were found in ARC and PVN, which are major players in the regulation of food intake [91]. This may be related to the timing of studying the genes or adaptations to weight loss.

## 5 Conclusion

Our study demonstrates that PYY(3–36) and Ex4 act synergistically, triggering multiple responses in both the brain and liver leading to increased leptin responsiveness, elevated plasma concentrations of SLR, and changes in gene regulation in the AP region of the brain. These findings likely contribute to underlying mechanisms to the superior effect on weight reduction. The significant increase in SLR levels may likely derive from cleavage of the overexpressed *Lepra* in the liver which we associate with improved leptin sensitivity as SLR's role in modulating circulating leptin levels is crucial for prober regulation of appetite, metabolism, and energy balance. Gene expression upregulation of *Cart*, *Pdyn, Bdnf* and *Klb* in the AP region specific to co-dosing PYY(3–36) and Ex4, also suggest enhanced leptin responsiveness and possibly indicates additional signalling through PYY(3–36) enhances Ex4's weight-lowering effects. These findings highlights that the effects cannot be attributed to a single gene or pathway, but rather an interplay of peripheral and central interactions, with leptin sensitivity holding a central role. Given that leptin resistance is a known factor in the development of obesity, yet still not fully understood, our findings on improved leptin responsiveness could represent underlying mechanisms important for obesity treatment. Therefore, we propose inclusion of leptin responsiveness assessments in clinical trials for anti-obesity medications by measuring SLR as it could serve as a biomarker of leptin responsiveness. Such assessments can deepen our knowledge of weight loss processes and improve the prospects for sustained weight control.

Finally, in the process of interpretating the data, we have contemplated several potential gaps in our understanding. These includes: 1) the complexity of the brain, with its variety of interconnected signals and compensatory mechanisms, 2) the lack of protein levels to confirm the gene expression findings 3) the reliance on SLR measurements taken four days post-leptin challenge in the interpretation of leptin sensitivity 4) understanding the role of SLR in the context of discontinued treatment to explore weight regain 5) extrapolation study findings from mice to humans. This transition always introduces a degree of uncertainty and presents a challenge for translation into clinical application. Nevertheless, it should be emphasized that the results of the study offer valuable insights and present an opportunity to develop new hypotheses, contributing to a deeper understanding of the intricate mechanisms involved in regulating energy balance and weight loss.

## Funding

This work was supported by Innovation Fond Denmark.

## Data availability statement

All data needed to evaluate the conclusions in the paper are present in the paper and/or the Appendix. Additional data related to this paper may be requested by contacting the corresponding author.

## CRediT authorship contribution statement

**Birgitte S. Wulff:** Writing – review & editing, Supervision. **Rune Ehrenreich Kuhre:** Writing – review & editing, Formal analysis. **Madhan Selvaraj:** Validation, Supervision, Software. **Jens F. Rehfeld:** Writing – review & editing, Supervision, Funding acquisition. **Kristoffer Niss:** Software, Formal analysis. **Johannes J. Fels:** Methodology. **Secher Anna:** Writing – review & editing, Supervision. **Kirsten Raun:** Writing – review & editing, Supervision. **Marina Kjaergaard Gerstenberg:** Writing – original draft, Validation, Software, Resources, Project administration, Methodology, Investigation, Funding acquisition, Formal analysis, Data curation, Conceptualization.

## Declaration of Competing interest

Novo Nordisk markets liraglutide and semaglutide for the treatment of diabetes and obesity. Marina Kjaergaard Gerstenberg, Rune Ehrenreich Kuhre, Madhan Selvaraj, Kristoffer Niss, Anna Secher and Kirsten Raun are full time employees at Novo Nordisk and stock owners through an employee offering program. Birgitte S. Wulff is retired from Novo Nordisk and owns Novo Nordisk stocks.
