# Supplementary material for: Improved leptin sensitivity and increased soluble leptin receptor concentrations may underlie the additive effects of combining PYY [and exendin-4 on body weight lowering in diet-induced obese mice
Source: Heliyon. 2024 Jun 3;10(12):e32009. doi: 10.1016/j.heliyon.2024.e32009 (PMC11341243; doi:10.1016/j.heliyon.2024.e32009)
Supplement: Multimedia component 1 [file mmc1.docx]

| Gene Symbol | Gene Name | Assay ID |
| --- | --- | --- |
| Ucp1 | uncoupling protein 1 | Mm01244861_m1 |
| Adrb3 | adrenergic receptor; beta 3 | Mm00442669_m1 |
| Nrg4 | neuregulin 4 | Mm00446254_m1 |
| Ngf | nerve growth factor | Mm00443039_m1 |
| Fgf21 | fibroblast growth factor 21 | Mm00840165_g1 |
| Pnpla1 | patatin-like phospholipase domain containing 1 | Mm01308771_m1 |
| Cpt1a | carnitine palmitoyltransferase 1a | Mm01231183_m1 |
| Gck | glucokinase | Mm00439129_m1 |
| Slc2a1 | solute carrier family 2 | Mm00441480_m1 |
| Cd36 | CD36 antigen | Mm00432403_m1 |
| Dgat1 | diacylglycerol O-acyltransferase 1 | Mm00515643_m1 |
| Slc27a1 | solute carrier family 27 | Mm00449511_m1 |
| Dio2 | deiodinase; iodothyronine; type II | Mm00515664_m1 |
| Gpbar1 | G protein-coupled bile acid receptor 1 | Mm04212121_s1 |
| Slc2a4 | solute carrier family 2 | Mm00436615_m1 |
| Acaca | acetyl-Coenzyme A carboxylase alpha | Mm01304257_m1 |
| Acacb | acetyl-Coenzyme A carboxylase beta | Mm01204671_m1 |
| Acox1 | acyl-Coenzyme A oxidase 1; palmitoyl | Mm01246834_m1 |
| Lpl | lipoprotein lipase | Mm00434764_m1 |
| Adcy3 | adenylate cyclase 3 | Mm00460371_m1 |
| Thrb | thyroid hormone receptor beta | Mm00437044_m1 |
| Thra | thyroid hormone receptor alpha | Mm00579691_m1 |
| Tacr1 | tachykinin receptor 1 | Mm00436892_m1 |
| Tacr2 | tachykinin receptor 2 | Mm01175997_m1 |
| Tacr3 | tachykinin receptor 3 | Mm00445346_m1 |
| Htr1a | 5-hydroxytryptamine (serotonin) receptor 1A | Mm00434106_s1 |
| Htr1b | 5-hydroxytryptamine (serotonin) receptor 1B | Mm00439377_s1 |
| Htr2b | 5-hydroxytryptamine (serotonin) receptor 2B | Mm00434123_m1 |
| Htr4 | 5 hydroxytryptamine (serotonin) receptor 4 | Mm00434129_m1 |
| Htr5a | 5-hydroxytryptamine (serotonin) receptor 5A | Mm00434132_m1 |
| Gapdh | glyceraldehyde-3-phosphate dehydrogenase | Mm99999915_g1 |
| Ppia | peptidylprolyl isomerase A | Mm02342430_g1 |
| Tbp | TATA box binding protein | Mm00446973_m1 |
| Ywhaz | tyrosine 3-monooxygenase/tryptophan 5-monooxygenase activation protein; zeta polypeptide | Mm01158417_g1 |
| Rps18 | ribosomal protein S18 | Mm02601777_g1 |
| Ppib | peptidylprolyl isomerase B | Mm00478295_m1 |
| Agrp | agouti related neuropeptide | Mm00475829_g1 |
| Cartpt | CART prepropeptide | Mm04210469_m1 |
| Npy | neuropeptide Y | Mm01410146_m1 |
| Npy2r | neuropeptide Y receptor Y2 | Mm01956783_s1 |
| Npy4r | neuropeptide Y receptor Y4 | Mm01220859_m1 |
| Pomc | pro-opiomelanocortin-alpha | Mm00435874_m1 |
| Mc3r | melanocortin 3 receptor | Mm00434876_s1 |
| Mc4r | melanocortin 4 receptor | Mm00457483_s1 |
| Sim1 | single-minded homolog 1 | Mm00441390_m1 |
| Bdnf | brain derived neurotrophic factor | Mm04230575_m1 |
| Crh | corticotropin releasing hormone | Mm04206019_m1 |
| Trh | thyrotropin releasing hormone | Mm01182424_mH |
| Hcrt | hypocretin | Mm01964030_s1 |
| Crhr1 | corticotropin releasing hormone receptor 1 | Mm00432670_m1 |
| Crhr2 | corticotropin releasing hormone receptor 2 | Mm00438308_m1 |
| Oxt | oxytocin | Mm01329577_g1 |
| Nts | neurotensin | Mm00481140_m1 |
| Ntsr1 | neurotensin receptor 1 | Mm00444459_m1 |
| Ntsr2 | neurotensin receptor 2 | Mm00435426_m1 |
| Insr | insulin receptor | Mm01211875_m1 |
| Glp1r | glucagon-like peptide 1 receptor | Mm00445292_m1 |
| Ghsr | growth hormone secretagogue receptor | Mm00616415_m1 |
| Gcg | glucagon | Mm00801714_m1 |
| Cck | cholecystokinin | Mm00446170_m1 |
| Cckbr | cholecystokinin B receptor | Mm00432329_m1 |
| Pdyn | prodynorphin | Mm00457573_m1 |
| Calca | calcitonin/calcitonin-related polypeptide; alpha | Mm00801462_m1 |
| Calcr | calcitonin receptor | Mm00432282_m1 |
| Ramp2 | receptor activity modifying protein 2 | Mm01292503_g1 |
| Sst | somatostatin | Mm00436671_m1 |
| Sstr2 | somatostatin receptor 2 | Mm00436684_m1 |
| Prkcd | protein kinase C; delta | Mm00440891_m1 |
| Nmu | neuromedin U | Mm00479868_m1 |
| Nmur2 | neuromedin U receptor 2 | Mm00600704_m1 |
| Grin1 | glutamate receptor; ionotropic; NMDA1 | Mm00433790_m1 |
| Grin2a | glutamate receptor; ionotropic; NMDA2A | Mm00433802_m1 |
| Grin2b | glutamate receptor; ionotropic; NMDA2B | Mm00433820_m1 |
| Grina | glutamate receptor; ionotropic; N-methyl D-aspartate-associated protein 1 | Mm00458208_m1 |
| Gria1 | glutamate receptor; ionotropic; AMPA1 | Mm00433753_m1 |
| Gria2 | glutamate receptor; ionotropic; AMPA2 | Mm00442822_m1 |
| Gria3 | glutamate receptor; ionotropic; AMPA3 | Mm00497506_m1 |
| Gria4 | glutamate receptor; ionotropic; AMPA4 | Mm00444754_m1 |
| Grik1 | glutamate receptor; ionotropic; kainate 1 | Mm00446882_m1 |
| Slc17a6 | solute carrier family 17 | Mm00499876_m1 |
| Grm5 | glutamate receptor; metabotropic 5 | Mm00690332_m1 |
| Syndig1 | synapse differentiation inducing 1 | Mm01271337_m1 |
| Syndig1l | synapse differentiation inducing 1 like | Mm01302876_m1 |
| Gad1 | glutamate decarboxylase 1 | Mm04207432_g1 |
| Gad2 | glutamic acid decarboxylase 2 | Mm00484623_m1 |
| Slc32a1 | solute carrier family 32 | Mm00494138_m1 |
| Gabbr2 | gamma-aminobutyric acid (GABA) B receptor; 2 | Mm01352554_m1 |
| Gabbr1 | gamma-aminobutyric acid (GABA) B receptor; 1 | Mm00444578_m1 |
| Gabrb1 | gamma-aminobutyric acid (GABA) A receptor; subunit beta 1 | Mm00433461_m1 |
| Gabrb2 | gamma-aminobutyric acid (GABA) A receptor; subunit beta 2 | Mm00433467_m1 |
| Gabrb3 | gamma-aminobutyric acid (GABA) A receptor; subunit beta 3 | Mm00433473_m1 |
| Syt1 | synaptotagmin I | Mm00436858_m1 |
| Syt2 | synaptotagmin II | Mm00436864_m1 |
| Syt3 | synaptotagmin III | Mm00443816_m1 |
| Syt7 | synaptotagmin VII | Mm00444502_m1 |
| Syt9 | synaptotagmin IX | Mm00502475_m1 |
| Th | tyrosine hydroxylase | Mm00447557_m1 |
| Slc6a3 | solute carrier family 6 | Mm00438388_m1 |
| Drd1 | dopamine receptor D1 | Mm01353211_m1 |
| Drd2 | dopamine receptor D2 | Mm00438545_m1 |
| Dbh | dopamine beta hydroxylase | Mm00460472_m1 |
| Comt | catechol-O-methyltransferase | Mm00514377_m1 |
| Socs3 | suppressor of cytokine signaling 3 | Mm01249143_g1 |
| Stat3 | signal transducer and activator of transcription 3 | Mm01219775_m1 |
| Creb1 | cAMP responsive element binding protein 1 | Mm00501607_m1 |
| Ptpn1 | protein tyrosine phosphatase; non-receptor type 1 | Mm00448427_m1 |
| Jak2 | Janus kinase 2 | Mm01208489_m1 |
| Irs2 | insulin receptor substrate 2 | Mm03038438_m1 |
| Mmp2 | matrix metallopeptidase 2 | Mm00439498_m1 |
| Gipr | gastric inhibitory polypeptide receptor | Mm01316344_m1 |
| Klb | klotho beta | Mm00473122_m1 |
| Fgfr1 | fibroblast growth factor receptor 1 | Mm00438930_m1 |
| Fosb | FBJ osteosarcoma oncogene B | Mm00500401_m1 |
| Fos | FBJ osteosarcoma oncogene | Mm00487425_m1 |
| Egr1 | early growth response 1 | Mm00656724_m1 |
| Npy1r | neuropeptide Y receptor Y1 | Mm04208490_m1 |
| Npy5r | neuropeptide Y receptor Y5 | Mm00443855_m1 |
| LEPR-TV1 | leptin receptor (leprb) | Taqman: NM_146146.2  SYBR-green: Forward Primer: CCTGGGCACAAGGACTGAAT  Reverse Primer: AGAAGAAGAGGACCAAATATCACTGATT |
| LEPR-TV3 | leptin receptor (lepra) | Taqman: NM_001122899.1  SYBR-green: Forward Primer: CCTGGGCACAAGGACTGAAT  Reverse Primer: GATTGGGTTCATCTGTAGTGGTCAT |
